# The Response of Macrophages and Neutrophils to Hypoxia in the Context of Cancer and Other Inflammatory Diseases

**DOI:** 10.1155/2016/2053646

**Published:** 2016-02-29

**Authors:** Antje Egners, Merve Erdem, Thorsten Cramer

**Affiliations:** Molecular Tumor Biology, Department of General, Visceral and Transplantation Surgery, RWTH University Hospital, 52074 Aachen, Germany

## Abstract

Lack of oxygen (hypoxia) is a hallmark of a multitude of acute and chronic diseases and can be either beneficial or detrimental for organ restitution and recovery. In the context of inflammation, hypoxia is particularly important and can significantly influence the course of inflammatory diseases. Macrophages and neutrophils, the chief cellular components of innate immunity, display distinct properties when exposed to hypoxic conditions. Virtually every aspect of macrophage and neutrophil function is affected by hypoxia, amongst others, morphology, migration, chemotaxis, adherence to endothelial cells, bacterial killing, differentiation/polarization, and protumorigenic activity. Prominent arenas of macrophage and neutrophil function, for example, acute/chronic inflammation and the microenvironment of solid tumors, are characterized by low oxygen levels, demonstrating the paramount importance of the hypoxic response for proper function of these cells. Members of the hypoxia-inducible transcription factor (HIF) family emerged as pivotal molecular regulators of macrophages and neutrophils. In this review, we will summarize the molecular responses of macrophages and neutrophils to hypoxia in the context of cancer and other chronic inflammatory diseases and discuss the potential avenues for therapeutic intervention that arise from this knowledge.

## 1. Introduction

Oxygen is of central importance for life and oxygen availability impacts on various physiological and pathophysiological processes across a wide range of species. To guarantee a sufficient supply of cells and tissues with O_2_, intricate oxygen delivery systems emerged during the evolution of biological complexity [1]. As the majority of organs and functional tissues display multicelled structures, local oxygen concentration is markedly different already in the healthy state. Indeed, local variances in O_2_ concentration are of central importance for embryonic development and normal organ function, for example, in cartilage, liver, and kidney [2, 3]. Lack of oxygen (hypoxia) is a hallmark of a multitude of acute and chronic diseases and, depending on degree and duration, can be either beneficial or detrimental for organ restitution and recovery [4]. The physiological differences in local oxygen concentration and the dynamic nature of oxygen in cells and tissues result in a wide range of oxygen partial pressure in mammalian organisms: values from 150 (lung apices), 100 (alveoli and arterial blood), to <20 mmHg (bone marrow) were reported [5]. In the context of inflammation, oxygen metabolism and, eventually, hypoxia are particularly important and significantly influence the course of inflammatory diseases. In this review, we will summarize how two prominent representatives of myeloid cells, macrophages and neutrophils, respond to hypoxia in the context of inflammation. We will focus on cancer and certain chronic inflammatory diseases. The intriguing importance of hypoxia/HIFs for myeloid cell function during infectious diseases has been covered by excellent reviews before [6, 7] and will not be discussed in detail here.

## 2. Inflammation and Hypoxia

The term “Inflammation” refers to a complex and highly ordered sequence of events by which the organism reacts to potentially harmful situations with the aim to defend and reconstitute tissue integrity. Inflammatory reactions can be triggered by microorganisms, chemicals, radiation, and mechanical force, to name a few. As chief effectors of the innate immune system, macrophages and neutrophils are of paramount importance in the inflammatory process and can be found in high numbers and strongly activated states in inflamed tissues. To fully comprehend the pivotal role of macrophages and neutrophils it is important to note that they not only engulf and kill microorganisms, but also orchestrate the activation of other cell types important for tissue/organ reconstitution, for example, lymphocytes, fibroblasts, and endothelial cells [8, 9]. Inflammation is intricately linked to oxygen metabolism [10]. Calor (heat), tumor (swelling), and rubor (redness), three of the four classical signs of inflammation, are based on enhanced blood flow and vascular permeability and are hence directly associated with altered oxygen distribution in inflamed areas. It is important to note that, while enhanced blood flow suggests boosted oxygen delivery, inflamed areas are usually severely hypoxic, most prominently in the acute stage [11–13]. Traditionally, this has been attributed to reduced oxygen diffusion due to higher interstitial pressure (swelling) and enhanced oxygen consumption of cells in their struggle to survive the harsh conditions of inflamed areas. Intriguing results from Campbell and colleagues have substantially expanded our perception of the mechanisms and functional relevance of hypoxia during inflammation in recent years [14]. While the neutrophil respiratory burst had been hypothesized to contribute to inflammation-associated hypoxia before, Campbell et al. presented convincing experimental evidence for a functional role of activated neutrophils in (a) oxygen depletion during colitis and (b) the induction of a transcriptional hypoxic response in intestinal epithelial cells [14]. Furthermore, mice with a defective respiratory burst (Nox2 −/− mice, a model system for chronic granulomatous disease) displayed severe impairment of inflammatory resolution in the gut, supporting the notion that hypoxia and hypoxia-induced transcriptional responses are functionally relevant for various aspects of the pathogenesis of inflammation [14]. The intricate link between hypoxia and inflammation is furthermore demonstrated by the observation that hypoxic conditions* per se* are able to induce inflammatory reactions [10]. Exposure of mice to 5% O_2_ for 60 minutes resulted in significantly enhanced protein expression of IL-6, TNF-*α*, and IL-1 in both serum and isolated macrophages [15]. Similar observations have been made in humans as healthy volunteers showed increased serum levels of proinflammatory factors after three overnight stays at high altitude [16]. The* in vitro* response of macrophages to hypoxia is complex and very much determined by macrophage phenotype and source as well as the culture conditions. In general, hypoxia exerts profound effects on various important aspects of macrophage biology, for example, expression of cell surface markers, viability, phagocytosis, metabolic activity, and cytokine release (comprehensively reviewed in [17]). The notion that ischemia-associated inflammatory reaction of lung and kidney grafts increases the risk of transplant failure and graft rejection demonstrates the clinical relevance of hypoxia-induced inflammation [18, 19]. All of the above translates into the possibility of a vicious circle where hypoxia and inflammation cooccur and mutually boost each other [20]. It is reasonable to assume that the molecular mechanisms that fine-tune the hypoxia-inflammation circle represent attractive targets for the treatment of chronic, nonresolving inflammation. The latter notion is further supported by alleviation of experimental colitis via delivery of oxygen [21, 22].

## 3. Hypoxia and Cells of the Innate Immune System

The history of research on the metabolism of immune cells resembles the history of cancer metabolism research as both topics were highly investigated in the beginning of the 20th century, followed by decades of faded interest and a surge in exciting and innovative results in the last 15 years (partly explained by unprecedented technical improvements and the widespread availability of omics methods). It was first reported a little over a century ago by Levene and Meyer that leukocytes display high glycolytic activity [23, 24]. This observation was confirmed by independent researchers in subsequent studies [25, 26] and led to the conclusion in 1938 that in leukocytes “fermentative metabolism was high in comparison to the oxidative metabolism and that splitting of sugar into lactic acid took place under aerobic conditions” [27]. As glycolysis represents the principal means to generate energy when oxygen is scarce, these findings argued for a pronounced dependence of leukocytes on the molecular mechanisms behind the response to hypoxia. While various transcription factors are induced upon oxygen depletion [28], hypoxia-inducible factors 1 and 2 (HIF-1, HIF-2, collectively termed HIFs) represent the principle molecular mediators of the hypoxic response [29, 30]. Genetic inactivation of HIF-1*α* in myeloid cells (via lysozyme M-Cre [31]) resulted in the notion that HIF-1*α* is indeed essential for inflammation in different acute and chronic murine model systems [32]. A similar genetic approach revealed that HIF-2*α* in macrophages is fundamental for proinflammatory cytokine expression upon LPS treatment as well as the* in vivo* response to cutaneous and peritoneal irritants [33]. These two fundamental studies established the functional importance of the HIFs for myeloid cell function and kicked off a huge number of follow-up studies that significantly broadened our understanding of the interplay between hypoxia and inflammation. The following chapters attempt to summarize parts of this work with special emphasis on the response of neutrophils and macrophages to hypoxia in the context of cancer and other inflammatory diseases.

## 4. Neutrophils

Neutrophilic granulocytes, or shortly neutrophils, are part of the mammalian innate immune system and recruited to wounds and infections during the early disease phase. With 50–70% they constitute the most abundant circulating white blood cell population. Chemical signals such as the chemokine IL-8, complement factor C5a, N-formylated peptides, platelet-activating factor, and leukotriene B4 attract neutrophils to inflammatory sites [34]. After sensing of bacteria or mediators of inflammation, neutrophils phagocytose microbes followed by assembly of an electron transport chain (NADPH oxidase) which shuttles electrons across the membrane to molecular oxygen for the generation of hypochlorous acid (HClO) and reactive oxygen species leading to lysis of microbes [35]. This process is termed “respiratory burst” and requires a significantly elevated consumption of molecular oxygen [36]. The respiratory burst represents an essential antimicrobial pathway of neutrophils. Furthermore, neutrophils can kill invading pathogens via release of granule contents, activating cytokines like TNF-*α*, IL-1, interferons, defensins, or reactive nitrogen species, and in some instances they generate extracellular traps [34]. As outlined in detail above, the presence of activated neutrophils at sites of inflammation results in oxygen depletion, a phenomenon aptly referred to as “inflammatory hypoxia,” underscoring the taut link between inflammation and hypoxia [37].

## 5. Neutrophils, Hypoxia, and Inflammation

Inflamed lesions often become severely hypoxic due to increased cellular oxygen demand and reduced availability caused by trauma, compression, or thrombosis [10–13]. Hypoxia and HIFs, in turn, influence various aspects of neutrophil biology. The rather short-lived naïve cells are activated and possess increased survival times within inflammatory environments [34]. Hypoxia-associated inhibition of neutrophil apoptosis was demonstrated to be NF-*κ*B-dependent, indicating NF-*κ*B as a regulator of the hypoxic response in neutrophils [38]. Furthermore, the neutrophil activating and survival factor MIP-1*β* (macrophage inflammatory protein-1*β*) was shown to be induced under hypoxic conditions, operating as an alternative mediator of neutrophil survival [38]. Neutrophil binding to the epithelium is facilitated by HIF-1-promoted *β*_2_ integrin expression [39]. Moreover, neutrophils mainly rely on high rates of glycolysis for the generation of ATP in which HIF-1*α* is critically involved by regulating the expression of key glycolytic enzymes [32]. The absence of HIF-1 causes depletion of intracellular ATP pools resulting in profound impairment of the inflammatory response due to decreased neutrophil aggregation, motility, bacterial killing, and invasion, once more suggesting HIF-1*α* to be crucial for neutrophil functionality [32, 40]. In addition, HIF-1 increases neutrophil expression of antimicrobial molecules, which is, for example, suggested by experiments showing that myeloid-specific HIF-1*α* deficiency increases susceptibility to local as well as systemic bacterial infections [32, 40]. Interestingly, neither neutrophil development nor differentiation is affected by specific deletion of HIF-1*α* in the myeloid progenitor lineage [32].

Far less is known about the role of HIF-2*α* during neutrophilic inflammation, although when isolated from patients they were shown to express increased amounts of HIF-2*α* [41]. Thompson et al. reported that HIF-2*α*-deficient murine inflammatory neutrophils displayed no impairment of chemotaxis, phagocytosis, or respiratory burst but elevated sensitivity to apoptosis leading to reduced neutrophilic inflammation [41]. In line with this notion, neutrophils carrying HIF-2*α* gain-of-function mutations had lower apoptosis rates. This study suggests a predominant role of HIF-2*α* for the resolution of inflammation. Certainly, further investigations of the functions of HIF-2 are needed to broaden our understanding of its influence on neutrophil performance during inflammation.

Hypoxia not only influences neutrophil activity; but neutrophils also shape the tissue microenvironment through depletion of local molecular oxygen. As they migrate across the epithelium they change the mRNA expression profile of epithelial cells, which consequently stabilize HIF and upregulate genes responding to hypoxia [14]. Infiltrating neutrophils further modulate the host response to inflammation, resulting in effective inflammatory resolution and tissue protection for which oxygen depletion proved to be critical [14]. Taken together, therapeutic targeting of neutrophils at inflammatory sites has to be carefully executed and precisely timed to prevent nonresolving inflammation or other potentially harmful outcomes.

## 6. Hypoxia and Tumor-Associated Neutrophils (TANs)

Neutrophils comprise a significant proportion of the inflammatory infiltrate in cancerous lesions and high levels of blood neutrophils were observed in patients suffering from advanced stage tumors [42]. In many cancer types, such as bronchoalveolar carcinoma [43], metastatic melanoma [42], and renal carcinoma [44], neutrophil accumulation was associated with poor prognosis, related to increased aggressiveness [45] or, as in human gliomas, to tumor grade [46]. In contrast, high neutrophil counts in gastric tumors correlate with favourable prognosis [47].

The potent influence of TANs on cancer development, progression, and outcome is becoming more and more appreciated [48–50]. In consideration of the prominent evidence for hypoxia affecting neutrophil behaviour and activity in tumors, it is surprising that until now only a small number of studies focused on this topic. Adherence of neutrophils to the endothelium, their activation, and elevated vessel extravasation leading to tumor infiltration were attributed to hypoxia-induced signalling in endothelial cells [51]. Furthermore, a report by Atai et al. suggests that HIF-1-dependent induction of osteopontin is crucial for the recruitment of neutrophils to neoplastic lesions [52]. IL-8, the main neutrophil attracting chemokine, is also induced in the course of the hypoxic response [53, 54]. Analysis of HIF-1-regulated, hypoxia-associated genes revealed augmented gene expression in TANs compared to splenic myeloid derived suppressor cells (MDSCs) for iNOS, IL-10, and IL-6 [55]. The neutrophil-specific serine protease elastase supports cancer cell proliferation [56] and its release is triggered under hypoxia [57]. Interestingly, in the hypoxic microenvironment of tumors, TANs are suggested to influence the classical (M1) versus the alternative (M2) polarization of macrophages [58, 59].

In analogy to the classification of macrophages, tumor-associated neutrophils were subdivided into two different polarization states: N1 and N2 [60]. Protumorigenic N2 TAN formation by TGF-*β*, which is another HIF-1 target and considered the master mediator of this process, was demonstrated to be induced under hypoxia [60]. In turn, TANs take on a proinflammatory and antitumorigenic N1 phenotype under conditions of TGF-*β* blockade and, by secreting reactive oxygen species, exhibit the potential to induce tumor cell lysis and growth arrest.

## 7. Neutrophils in Other Inflammatory Diseases

Macrophages were for a long time attributed to be the central players during inflammatory disease like rheumatoid arthritis (RA), whereas the influence of neutrophils in this context was largely elusive. However, compared to macrophages neutrophils are often found at much higher numbers at inflammatory sites and they are similarly capable to present antigens to and activate T-cells [61]. Neutrophils represent the most abundant immune cell type in the synovial fluid from joints of RA patients and were found at active sites of bone and cartilage destruction in this setting [62, 63]. Elevated secretion of ROS by neutrophils was suggested to be part of the disease driving processes during RA progression [64].

In the course of inflammatory liver disease, neutrophils were directly implicated in hepatocellular death mediated by the respiratory burst. They were shown to be recruited through TNF-*α* and other factors released by tissue-resident Kupffer cells [65]. Even in cases where other stimuli led to destruction of the liver parenchyma, the involvement of neutrophils often aggravated disease outcome [66]. Ischemia-reperfusion liver injury taking place, for example, during transplantation is another type of inflammatory process in which primed neutrophils take part to a significant extent [67, 68]. Furthermore, as a consequence of extensive alcohol consumption, neutrophil influx into the liver, hepatocyte degeneration, and necrosis finally result in neutrophilic steatohepatitis [69, 70]. Mechanistically, osteopontin was suggested to be critically involved as it is induced in rat hepatocytes after feeding the animals with an ethanol-containing liquid diet and its cleaved form correlated with neutrophil infiltration [71].

In patients suffering from chronic obstructive pulmonary disease (COPD), neutrophils are the most abundant inflammatory cells in the bronchial wall and lumen and neutrophil accumulation was reported to correlate with the decline of lung functionality [71–74]. In response to pollutants or infective agents, pulmonary epithelial cells or resident alveolar macrophages secrete chemoattractants inducing the recruitment of neutrophils and other immune cells [75]. However, not only elevated tissue invasion but also impaired neutrophil clearance was implicated in the pathogenesis of COPD as alveolar macrophages exhibit a loss in phagocytic activity and cigarette smoke has directly been linked to reduced phagocytosis of apoptotic neutrophils [76, 77]. Clinical trials investigating the efficiency of drugs that promote neutrophil apoptosis and clearance in patients with chronic obstructive pulmonary disease are ongoing.

## 8. Macrophages

Macrophages are phagocytic cells and crucial effectors of innate immunity in the primary response to pathogens besides their key role in acute and chronic inflammatory responses. Many pathological processes with macrophage involvement (e.g., inflammation, wound healing, atherosclerosis, and tumors) are characterized by hypoxia [29]. Hypoxic zones arise especially in inflamed tissues, driving cellular metabolism to adapt to this hostile microenvironment. It is therefore not surprising to note that HIFs are found stabilized in macrophages at various stages of activation and polarization [78, 79] and that inhibition of HIF impacts on a plethora of archetypical macrophage functions such as aggregation, migration, and invasion [6, 32, 40].

## 9. Response to Hypoxia: Macrophages and HIFs

Hypoxia influences various aspects of macrophage function, including energy metabolism and different immune responses. Myeloid cell-specific inactivation of HIF-1*α* via Cre/loxP-mediated conditional gene inactivation resulted in notably reduced inflammatory responses in skin and joint inflammation [32]. In this experimental setting, significantly reduced intracellular ATP levels were detected in HIF-1*α*-deficient macrophages, enforcing the pivotal importance of HIF-1*α*-controlled glycolysis for energy generation in myeloid cells [24, 25]. Besides sterile inflammation, hypoxia also commonly occurs in areas of infection [6]. As macrophages are of paramount importance in the first line defence against invasive microorganisms, it has long been hypothesized that these cells must be especially equipped to cope with and function in hypoxic areas. It was convincingly shown, again via conditional gene ablation in mice, that HIF-1*α* is of paramount importance for bacterial killing activity of macrophages (and neutrophils) [32, 40]. Of note, the antimicrobial effect of HIF-1*α* was not limited to hypoxic culture conditions, but clearly evident under ambient air, further supporting the above outlined hypoxia-independent importance of HIF-1*α* in macrophages. In line with this notion, bacterial infection of macrophages under normoxic culture conditions results in robust stabilization of HIF-1*α* protein [40]. This effect is (partly) mediated by lipopolysaccharide (LPS, a component of the outer membrane of gram-negative bacteria) as LPS represents a potent inducer of both mRNA expression and HIF-1*α* protein accumulation in murine macrophages and human monocytes [80, 81]. This suggested a functional importance of toll-like receptor 4 (TLR-4, the archetypical LPS receptor) for HIF-1*α* activation and resulted in the publication of a plethora of interesting publications addressing this point. We know today that HIF-1*α* and TLRs interact bidirectionally on many biologically relevant levels [82]. On the one hand, HIF-1*α* regulates the surface expression of various TLRs (e.g., TLR-2, -4, -6, and -9) [83–85]. On the other hand, intracellular signal transduction of several TLRs is (partially) mediated by HIF-1*α* (e.g., TLR-2, -3, -4, -7/8, and -9) [85–88]. Of special interest in downstream TLR signalling is the NF-*κ*B family of transcription factors. NF-*κ*B represents a pivotal control element of the immune system and is potently induced by LPS [89]. Of note, LPS-induced HIF-1*α* activation is dependent on NF-*κ*B in human monocytes and murine macrophages [80, 90]. Taken together, the paramount importance of HIF-1*α* for TLR activation qualifies as a molecular explanation for the outlined function of HIF-1*α* in microorganism defence.

Compared with the vast amount of literature available regarding the importance of HIF-1*α*, the role of HIF-2*α* for the hypoxic response of macrophages is only beginning to emerge. Hypoxic culture conditions lead to robust accumulation of HIF-2*α* protein in various myeloid cell types, for example, human monocyte-derived macrophages (MDM) and primary murine bone marrow-derived macrophages (BMDM) [33, 91]. Functional inactivation of HIF-2*α*, by either RNA interference in MDM or Cre/loxP-mediated deletion in BMDM, resulted in significantly reduced transcriptional responses to hypoxia (and to proinflammatory stimulation with LPS plus interferon-*γ*) [33, 78]. Comparable to other cell types, the function of HIF-1*α* and -2*α* for the hypoxic response of macrophages is not redundant at all times, but distinct regarding the regulation of selected factors [30]. For example, loss of HIF-2*α* in macrophages does not impact on the expression of two classical HIF-1*α* target genes, the inducible NO synthase (iNOS) and VEGF-A [33, 92]. On the other hand, HIF-2*α* activates soluble VEGF receptor-1, a potent inhibitor of VEGF, while HIF-1*α* is without effect [92].

## 10. Macrophage Polarization and Arginine Metabolism

Macrophages are highly plastic cells and can rapidly change their polarization in response to microenvironmental cues [93]. In recent years, the concept of a Th1-driven, proinflammatory macrophage (termed M1) and a Th2-driven, proangiogenic/immune-evasive M2 macrophage has evolved. The above outlined connection of hypoxia and inflammation led to the question how/if HIFs are involved in macrophage plasticity. Takeda and colleagues were the first to address this point and intriguingly found that HIF-1*α* and HIF-2*α* contributed to macrophage polarization in opposing ways [94]. While Th1 cytokine-induced M1 skewing of murine BMDM is paralleled by HIF-1*α* protein stabilization, M2 induction with interleukin-4 resulted in HIF-2*α* protein accumulation [94]. While hypoxia potentiated this response, the cytokine-induced HIF stabilization was clearly detectable under normoxic conditions, further supporting the notion of hypoxia-independent HIF accumulation. Analysis of HIF-1*α*- or HIF-2*α*-deficient murine macrophages further strengthened the opposing roles of the 1*α* and 2*α* isoform for macrophage polarization [94]. It is important to note that macrophage polarization was not the focus of the experimental setup applied by the Johnson group as their primary goal was to unravel the role of HIFs for NO homeostasis in macrophages. In principle, two factors determine extracellular NO abundance (via competition for the precursor L-arginine): the family of NO synthases (most prominently iNOS) produces NO while arginase-1 metabolizes L-arginine into ornithine and polyamines, effectively reducing extracellular NO levels [95]. Interestingly, these factors are differently expressed in polarized macrophages: iNOS in M1 and arginase-1 in (murine) M2 macrophages [93, 96]. Via the identification of arginase-1 as a HIF-2*α* target gene, Takeda et al. provided a molecular mechanism for the opposing effect of the HIFs on macrophage polarization. It was first reported in the late 1980s that activated murine macrophages are able to kill tumor cells via iNOS-derived NO [97, 98]. The rapid progression of the majority of malignant tumors led to the assumption that macrophage-mediated killing is somehow compromised during malignant progression [95]. Indeed, it could be shown that NO production in many tumors is reduced due to diminished iNOS activity in macrophages [99]. Convincing data from independent research groups argue for a time- or stage-dependent effect: at early stages, (M1) macrophage-derived NO results in tumor cell killing. Dying tumor cells release various factors (e.g., TGF-*β*, interleukin-10, or sphingosine-1-phosphate) that lead to M2 polarization of macrophages, which express high levels of arginase-1, ultimately resulting in reduced intratumoral NO abundance, thus contributing to tumor progression [95]. The work by Takeda et al. complements as well as expands this concept by pointing to HIF-2*α* as an important molecular mechanism in the switch from M1 to M2 during tumor progression. Using spheroids from human breast cancer cells, Werno and colleagues presented additional experimental evidence for a role of HIF-1*α* for M1 polarization [100].* In vivo* confirmation of these results is missing thus far, but as more and more reliable antibodies against M1/M2-specific markers are available and FACS-based characterization of the tumor immune infiltrate prevails this should only be a matter of time.

## 11. Tumor-Associated Macrophages (TAMs)

Hypoxia is a hallmark of solid tumor formation and a potent driver of the malignant phenotype. In certain entities, cervical, breast, prostate, and head and neck cancer as well as melanoma, hypoxia represents an independent prognostic factor [101]. Macrophages are attracted by and accumulate in hypoxic regions and intratumoral hypoxia is a pivotal regulator of TAM function [102, 103]. Among these, angiogenesis induction is probably the best studied phenomenon. In human breast cancer, the proangiogenic factor VEGF-A is expressed almost exclusively in macrophages in hypoxic areas, a process largely dependent on HIF-1*α*, as suggested by experiments performed with murine macrophages [32, 104]. In addition to VEGF-A, the expression of additional proangiogenic molecules in TAMs like basic fibroblast growth factor (bFGF), CXCL8/IL-8, adrenomedullin, and matrix metalloproteinase 9 (MMP-9) is induced by hypoxia in a HIF-1*α*-dependent manner [105]. Furthermore, HIF-1 activity was shown to enhance the expression of the chemokine CXCL12 and of its receptor CXCR4, both crucially involved in angiogenesis and cancer metastasis [106]. Angiogenesis inhibitors were among the first molecular targeted drugs approved for cancer therapy and their market launch was paralleled by enormous expectations. Unfortunately, initial enthusiasm soon dissipated as the antiproliferative efficacy did not meet the anticipations [107, 108]. The demonstration of enhanced hypoxia and HIF stabilization in rodent tumor models upon application of angiogenesis inhibitors lead to the assumption that HIFs are causally involved in the resistance to antiangiogenic therapy [109]. It was subsequently shown by various groups that inhibition of HIF-1 was able to enhance the efficacy of angiogenesis inhibitors [110–112]. Clinical studies are under way to confirm these observations in patients with advanced cancers [113]. Besides angiogenesis, TAMs are able to fuel cancer progression via their suppressive effect on adaptive immunity [114]. Macrophages play a crucial role in this setting as they can inhibit T cell-mediated tumor cell killing in a hypoxia/HIF-1*α*-dependent manner.

Compared to the available information on HIF-1*α*, the importance of HIF-2*α* for TAM biology has been explored to a far lesser extent. An immunohistochemical study with human breast cancer samples displayed HIF-2*α* protein in TAMs and reported a correlation between high TAM HIF-2*α* and tumor vascularity and tumor grade [115]. The same group showed HIF-2*α*-positive TAMs in human head and neck squamous carcinoma, albeit without association with clinical parameters [116]. Celeste Simon and coworkers used conditional gene targeting to address the functional importance of HIF-2*α* in TAMs. Myeloid-specific loss of HIF-2*α* resulted in reduced numbers of TAMs in two murine model systems (DEN-induced liver tumors and inflammation-associated intestinal tumors (via AOM+DSS)) [33]. The authors identified reduced migratory and invasive ability of macrophages as the underlying mechanisms. Interestingly, intracellular ATP levels were not affected, in contrast to macrophages displaying a functional loss of HIF-1*α* [32]. Murine intestine is currently the best studied organ with respect to the role of HIF-2*α* in inflammatory and proliferative conditions and the existing data were comprehensively summarized in a recent review [117].

## 12. Macrophages in Other Inflammatory Diseases

Rheumatoid arthritis (RA) is a chronic inflammatory joint disease that causes bone and cartilage destruction. Hypoxia occurs in the course of RA in synovial tissues, potentially affecting inflammation, angiogenesis, synovial responses, and resolution [118, 119]. Increased HIF-1*α* protein levels were detected in macrophages of RA patients [120] and myeloid-specific deletion of HIF-1*α* reduces joint swelling and inflammatory activity in a murine arthritis model [32].

Despite impressive improvements in therapy and prevention, cardiovascular diseases are still the leading cause of death in developed countries. Atherosclerosis is a pivotal process in the pathogenesis of cardiovascular diseases and inflammation is centrally involved in atherosclerosis development. Interestingly, a causal role for hypoxia in the pathogenesis of atherosclerotic plaque formation was hypothesized more than 60 years ago [121]. Indeed, while the healthy arterial media is already characterized by reduced oxygen partial pressure (20–50 mmHg), even lower values have been measured in atherosclerotic plaques [122, 123]. Macrophages with ingested lipids (foam cells) are a histopathological hallmark of atherosclerotic plaques. Numerous studies have analyzed the role of HIF-1*α* in macrophages and foam cells for processes important in atherosclerosis development (reviewed in [124]). While the majority of these studies suggest a functional importance of HIF-1*α*,* in vivo* studies with macrophage-specific HIF1*α* null mice have failed to confirm this notion. Unfortunately, the results of these studies have thus far only been presented on scientific conferences and not as peer-reviewed publications. Hence, a causal role of HIF-1*α* in macrophages for the pathogenesis of atherosclerosis remains elusive at this time.

## 13. Potential Therapeutic Implications

The protumorigenic role of hypoxia, the functional connection of HIFs with “cancer genes,” and the observation that the majority of HIF-regulated biological pathways are positively associated with the malignant phenotype made the HIFs an attractive target for drug development [125]. Currently, 75 studies are listed in https://www.clinicaltrials.gov/ that aim to analyze the efficacy of HIF-1 inhibitors for a wide spectrum of diseases, for example, cancer, wound healing, and cardiovascular diseases. As outlined above, anticancer molecular targeted drugs were thus far not able to meet the gigantic expectations associated with their approval. Against this background it would not be surprising to observe resistance against and, subsequently, diminished antiproliferative efficacy of HIF-targeting substances in the clinical setting. Indeed, we and others have shown that cancer cells are able to compensate for the loss of HIFs very effectively [126]. We therefore strongly believe that it is of crucial importance to analyze the mechanisms that underlie resistance/compensation towards/of HIF inhibition in order to identify combination partners with the potential to result in long-lasting, effective, and well tolerable HIF-based cancer therapy.

## 14. Perspective

Hypoxia is a hallmark of the hostile microenvironment of inflammation and macrophages and neutrophils, the chief effectors of innate immunity, have evolved to cope with and function in these conditions. Albeit several oxygen-sensitive transcription factors have been described, tissue-specific knock-out mouse models have enabled in-depth deconstruction of the hypoxic response, demonstrating that HIFs are absolutely essential for proper myeloid cell function under hypoxic conditions. Compared with the large amount of literature on the role of HIF-1*α*, the functional importance of HIF-2*α* remains elusive for several inflammatory conditions. Another key question is how effective HIF-modifying agents will prove to be in the therapy of acute and chronic inflammation and what kind of side effects will emerge. Will resistance against HIF inhibitors result in diminished antiproliferative or anti-inflammatory efficacy over time and will we be able to deconstruct the underlying mechanisms to design smart combination therapies? These questions, among others, will have to be addressed in order to achieve a successful translation of the exciting science that we had the pleasure to witness after the initial publication of HIF-1 in 1992 and of HIF-2 five years later.
